# Identification and characterization of the ergochrome gene cluster in the plant pathogenic fungus *Claviceps purpurea*

**DOI:** 10.1186/s40694-016-0020-z

**Published:** 2016-03-22

**Authors:** Lisa Neubauer, Julian Dopstadt, Hans-Ulrich Humpf, Paul Tudzynski

**Affiliations:** 1 Institute of Plant Biology and Biotechnology, Westfälische Wilhelms-Universität Münster, Schlossplatz 8, 48143 Münster, Germany; 2 Institute of Food Chemistry, Westfälische Wilhelms-Universität Münster, Corrensstr. 45, 48149 Münster, Germany

**Keywords:** *Claviceps purpurea*, Secondary metabolism, Biosynthesis, Gene cluster, Ergot pigments, Ergochromes, Anthraquinones, Nonreducing polyketide synthase

## Abstract

**Background:**

*Claviceps purpurea* is a phytopathogenic fungus infecting a broad range of grasses including economically important cereal crop plants. The infection cycle ends with the formation of the typical purple-black pigmented sclerotia containing the toxic ergot alkaloids. Besides these ergot alkaloids little is known about the secondary metabolism of the fungus. Red anthraquinone derivatives and yellow xanthone dimers (ergochromes) have been isolated from sclerotia and described as ergot pigments, but the corresponding gene cluster has remained unknown. Fungal pigments gain increasing interest for example as environmentally friendly alternatives to existing dyes. Furthermore, several pigments show biological activities and may have some pharmaceutical value.

**Results:**

This study identified the gene cluster responsible for the synthesis of the ergot pigments. Overexpression of the cluster-specific transcription factor led to activation of the gene cluster and to the production of several known ergot pigments. Knock out of the cluster key enzyme, a nonreducing polyketide synthase, clearly showed that this cluster is responsible for the production of red anthraquinones as well as yellow ergochromes. Furthermore, a tentative biosynthetic pathway for the ergot pigments is proposed. By changing the culture conditions, pigment production was activated in axenic culture so that high concentration of phosphate and low concentration of sucrose induced pigment syntheses.

**Conclusions:**

This is the first functional analysis of a secondary metabolite gene cluster in the ergot fungus besides that for the classical ergot alkaloids. We demonstrated that this gene cluster is responsible for the typical purple-black color of the ergot sclerotia and showed that the red and yellow ergot pigments are products of the same biosynthetic pathway. Activation of the gene cluster in axenic culture opened up new possibilities for biotechnological applications like the dye production or the development of new pharmaceuticals.

**Electronic supplementary material:**

The online version of this article (doi:10.1186/s40694-016-0020-z) contains supplementary material, which is available to authorized users.

## Background

The biotrophic ascomycete *Claviceps purpurea* infects a broad range of grasses including economically important cereal crop plants like rye, wheat and barley [1]. The fungus infects exclusively the young ovaries of the host plants. After successful colonization the ovary is replaced by fungal mycelium and production of conidia begins. The infection cycle ends with the formation of a sclerotium, the resting structure of the fungus [2, 3]. Ergot alkaloids, the best characterized secondary metabolites of *C. purpurea*, are produced exclusively in the sclerotial tissue. These toxins are historically important as they affect the central nervous system of mammalians and were the reason for severe intoxications in the past, caused by consumption of contaminated bread [4]. Sclerotia are shaped like a grain but are usually larger (2–25 mm) and the hard outer cortex is pigmented purple-black. Sclerotia contain 1–2 % (w/w) of pigments belonging to different structural groups [5].

**Fig. 1:**
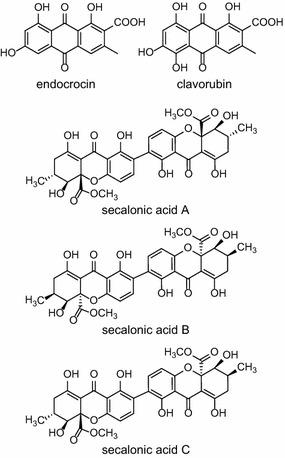
Ergot pigments. Endocrocin and clavorubin are red hydroxyanthraquinonecarboxylic acids. Secalonic acids A, B and C belong to the group of yellow ergochromes

The red pigments endocrocin and clavorubin (Fig. 1) are anthraquinone derivatives [6]. Both are hydroxyanthraquinonecarboxylic acids and differ only in one hydroxy group. Clavorubin is red whereas endocrocin has an orange-red color.

The yellow ergochromes are dimers of tetrahydroxanthone units [7]. Four different xanthone derivatives were described as ergochrome units in *C. purpurea* and all possible combinations of two of these units occur in nature [8]. Their concentration in the sclerotia is considerably higher (5 g/kg) than that of endocrocin and clavorubin (40 mg/kg) [8]. Ergochromes are known for their biological activity. Many of them show anti-inflammatory, cytostatic and anti-tumor activity or a neuroprotective effect [9–11]. Due to the structural similarity of the ergochromes to the anthraquinone pigments (Fig. 1) it is likely that both pigments are products of the same biosynthetic pathway. In *Aspergillus nidulans* it has already been shown that endocrocin is a shunt product during the production of xanthones [12, 13]. Nevertheless, the biosynthesis of the ergochromes in *C. purpurea* remains unclear, although some intermediates like emodin have been described [8].

Endocrocin could be identified in extracts of various other fungi [14–16] but its biosynthesis is best characterized in *Aspergillus fumigatus* where a cluster of four genes has been identified [17]. The key enzyme of the cluster is a nonreducing polyketide synthases (NR-PKS) lacking the thioesterase (TE) domain usually necessary for releasing the polyketide product from the enzyme. In these special types of NR-PKSs the polyketide is released from the PKS by a metallo-β-lactamase-type thioesterase (MβL-TE) [18]. Recently, in *A. fumigatus* a second gene cluster has been identified which also contributes to the formation of endocrocin as a shunt product from production of the anthraquinone-derivative trypacidin [19]. Both clusters show homologies to clusters responsible for the formation of related anthraquinone-derivatives such as geodin in *Aspergillus terreus* [20] and the xanthones in *A. nidulans* [12, 13].

This paper reports the identification of a gene cluster in *C. purpurea* which shows high homology to these gene clusters and is involved in ergochrome biosynthesis. Besides the classical ergot alkaloid cluster, this is the first functional analysis of a secondary metabolite gene cluster in the ergot fungus.

## Results

### Characteristics of the gene cluster

**Fig. 2:**
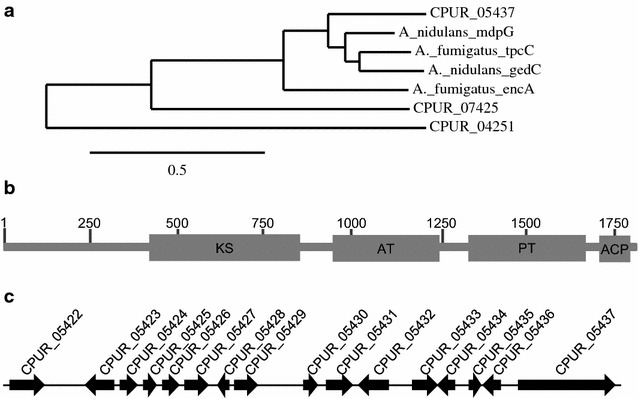
**a** Phylogenetic analysis of the three NR-PKS of *C. purpurea* and homologs from other fungi. Sequences were aligned with MUSCLE (v3.8.31) and the phylogenetic tree was constructed with the maximum likelihood method using Phylogeny.fr [54]. **b** Domain structure of the nonreducing polyketide synthases CPUR_05437. *KS* ketosynthase, *AT* acyltransferase, *PT* product template, *ACP* acyl carrier protein. **c** Organization of the putative pigment gene cluster. Orientation of the *arrows* indicates the direction of transcription

Bioinformatic analysis of the *C. purpurea* genome revealed the presence of three nonreducing polyketide synthases [21]. In a phylogenetic tree CPUR_05437 groups with NR-PKSs of the *A. terreus* geodin cluster (GedC) [20], the *A. fumigatus* trypacidin (TpcC) [19] and endocrocin (EncA) [17] clusters and the *A. nidulans* monodictyphenone cluster (MdpG) [12] (Fig. 2a). Analysis of the domain structure of this protein, using the NCBI Conserved Domain Database, shows that CPUR_05437 also belongs to the group of TE-less PKSs (Fig. 2b) and the genes surrounding *CPUR_05437* (Fig. 2c) have significant identity to the genes of the endocrocin, monodictyphenone, trypacidin, and geodin biosynthetic clusters (Table 1). Particularly the genes probably encoding enzymes necessary for the formation of endocrocin are present in the *C. purpurea* cluster, for example the MβL-TE type hydrolase *CPUR_05436* and the decarboxylase *CPUR_05435*. Homologs to other genes involved in biosynthesis of monodictyphenone, trypacidin, or geodin are also located in the *C. purpurea* gene cluster. However, for some genes no homolog could be identified in *C. purpurea*, and three genes (*CPUR_05425*, *CPUR_05426*, *CPUR_05431*) appear to be *Claviceps* specific.

**Table 1:** Predicted functions and homologs of the pigment gene cluster in *C. purpurea*

*C. purpurea*	Enc	MDP	Tpc	Ged	Predicted function
*CPUR_05423*	–	*mdpD*	–	–	Monooxygenase
*CPUR_05424*	–	–	*tpcM*	*gedG*	Methyltransferase
*CPUR_05425*	–	–	–	–	?
*CPUR_05426*	–	–	–	–	?
*CPUR_05427*	–	*mdpL*	*tpcI*	*gedK*	Oxidase
*CPUR_05428*	–	*mdpB*	–	–	Dehydratase
*CPUR_05429*	–	*mdpC*	–	–	Reductase
*CPUR_05430*	–	*mdpK*	*tpcG*	*gedF*	Oxidoreductase
*CPUR_05431*	–	–	–	–	Monooxygenase
*CPUR_05432*	–	*mdpA*	*tpcD*	*gedD*	Coactivator
*CPUR_05433*	–	*mdpE*	*tpcE*	*gedR*	Transcription factor
*CPUR_05434*	–	*mdpH*	*tpcL*	*gedH*	Decarboxylase
*CPUR_05435*	*encC*	*mdpH*	*tpcK*	*gedl*	Decarboxylase
*CPUR_05436*	*encB*	*mdpF*	*tpcB*	*gedB*	Hydrolase
*CPUR_05437*	*encA*	*mdpG*	*tpcC*	*gedC*	Non-reducing PKS
–	–	*mdpI*	–	–	Acyl-CoA synthase
–	–	*mdpJ*	*tpcF*	*gedE*	Glutathione *S*-transferase
–	–	–	*tpcA*	*gedA*	*O*-Methyltransferase
–	–	–	*tpcJ*	*gedJ*	Dihydrogeodin oxidase
–	–	–	–	*gedL*	Sulochrin halogenase
–	–	–	*tpcH*	–	Methyltransferase

*Enc* endocrocin *A. fumigatus* [17], *MDP* monodictyphenone *A. nidulans* [12], *Tpc* Trypacidin *A. fumigatus* [19], *Ged* Geodin *A. terreus* [20]

### *In planta* expression

**Fig. 3:**
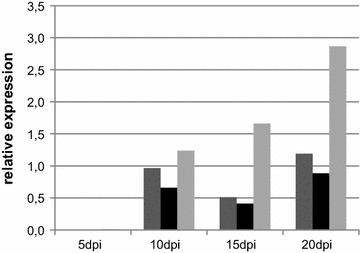
*In planta* expression of the central cluster gene *CPUR_05436*. Three independent experiments are shown in the *C. purpurea* wild type 5, 10, 15 and 20 days post-infection (dpi). Expression levels were determined by qRT-PCR and normalized against the housekeeping genes β-tubulin, γ-actin, and glyceraldehyde-3-phosphate dehydrogenase

To check if the gene cluster is responsible for the typical pigment formation of *C. purpurea*, we first tested if the expression of a central cluster gene (*CPUR_05436*) is correlated with the pigmentation *in planta*. Therefore, rye plants were infected with the *C. purpurea* wild type strain Ecc93 and the relative expression of *CPUR_05436* was quantified via quantitative reverse-transcription PCR (qRT-PCR) 5, 10, 15 and 20 days post-infection (dpi). Detectable gene expression starts 10 dpi and shows an increase towards the late stages of infection (Fig. 3). A correlation with the formation of the sclerotia and thus the pigment production could be observed. Although the results show high variability due to the heterogenous biological material (the infection process cannot be completely synchronized), this tendency was consistent with results from three biological replicates.

### Gene expression in axenic culture and optimization of the culture conditions

**Fig. 4:**
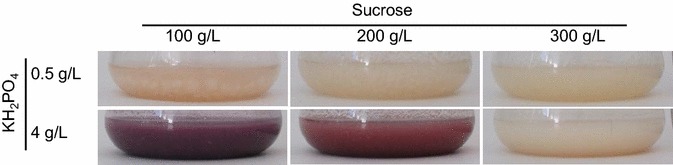
Optimization of the culture conditions. Pigmentation of *C. purpurea* liquid cultures depends on the sucrose and phosphate concentration. Cultures were grown for 10 days

**Fig. 5:**
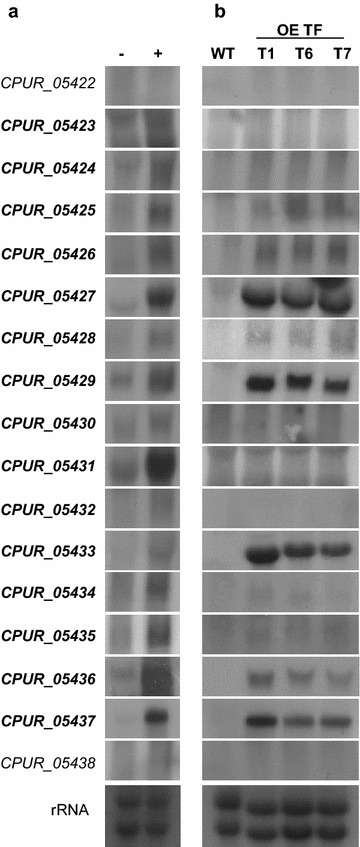
Co-regulation of the pigment cluster genes. **a** The *C. purpurea* wild type strain was grown for 10 days under conditions not favorable (−) and favorable (+) for pigment production. (−) 0.5 g/L KH_2_PO_4_ and 300 g/L sucrose; (+) 4 g/L KH_2_PO_4_ and 100 g/L sucrose. **b** Regulation of the pigment cluster genes by a zinc finger transcription factor. The wild type as well as three independent *CPUR_05433* overexpression mutants were grown for 7 days in under conditions unfavorable for the pigment production (300 g/L sucrose and 0.5 g/L KH_2_PO_4_)

Under standard laboratory conditions, using liquid cultures with BII complex medium, the gene cluster was not expressed and no coloration of the culture broth was visible. By using a defined medium with different sucrose concentrations (100, 200, 300 g/L), the gene cluster became activated in the cultures with a low sucrose concentration of 100 g/L. After 10 days of cultivation first signs of pigmentation occurred and after 14 days red pigmentation of the culture became evident. Additional variation in phosphate by adding different amounts of KH_2_PO_4_ led to marked pigmentation of the cultures at the lower concentration of sucrose (100 or 200 g/L) and the higher concentration of phosphate (4 g/L KH_2_PO_4_) already after 10 days of cultivation (Fig. 4). As shown by northern blot analysis (Fig. 5a), the pigment formation correlates in a qualitative sense with the expression of the predicted cluster genes *CPUR_05423* to *CPUR_05437*. Genes *CPUR_05422* and *CPUR_05438* are not co-regulated with the cluster genes, confirming the borders of the gene cluster. Taken together, there is an inverse influence of sucrose and phosphate concentration on pigment expression and an activation of the pigment production in axenic culture is possible by supplying high amounts of phosphate in combination with low sucrose concentrations.

### Overexpression of the transcription factor and the PKS

To verify if the PKS CPUR_05437 gene cluster is responsible for the pigment synthesis in *C. purpurea* we overexpressed the Zn_2_Cys_6_ transcription factor *CPUR_05433* by introducing an additional copy under the control of the strong constitutive *A. nidulans* oliC (mitochondrial ATP synthase subunit 9 gene) promoter (Additional file 1: Figure S1A). For three independent transformants the overexpression of the transcription factor as well as of nine further cluster genes could be confirmed by northern blot analyses (Fig. 5b). These results show that the genes *CPUR_05425*, *CPUR_05426*, *CPUR_05427*, *CPUR_05428*, *CPUR_05429*, *CPUR_05434*, *CPUR_05435*, *CPUR_05436*, as well as *CPUR_05437* are regulated by CPUR_05433. As expression of the other cluster genes is very low, the co-regulation by the transcription factor could not be confirmed by northern analyses.

**Fig. 6:**
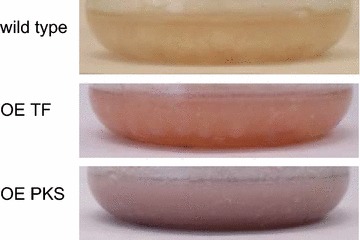
Overexpression of the transcription factor CPUR_05433 and the PKS CPUR_05437 respectively, leads to pigment formation. All strains were grown under conditions unfavorable for the pigment production (300 g/L sucrose and 0.5 g/L KH_2_PO_4_)

Nevertheless, the transcription factor overexpression mutants (OE TF) show a clear orange-red pigmentation, also under growth conditions unfavorable for pigment production (Fig. 6). To analyze the products of the gene cluster, pigments were extracted from fungal mycelia and the metabolite profile of the wild type and OE TF mutants was compared using reverse-phase–high-performance liquid chromatography–diode-array detection–high resolution mass spectrometry (RP–HPLC–DAD–HRMS). Identification of the pigments was based on three parameters, the characteristic UV-spectra [16, 22], the exact mass (±1.5 ppm) and subsequent HMRS^n^ experiments. The latter were compared to already described fragments in the literature [12, 16].

**Fig. 7:**
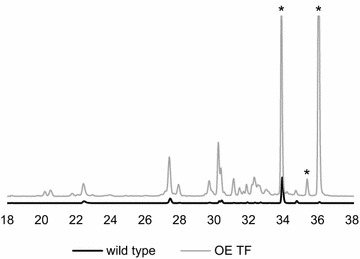
Comparison of the UV spectra at 332 nm between the wild type and OE TF strain. *Asterisks* indicate the yellow appearing metabolites which are produced in considerably higher concentrations when the transcription factor *CPUR_05433* is overexpressed

**Fig. 8:**
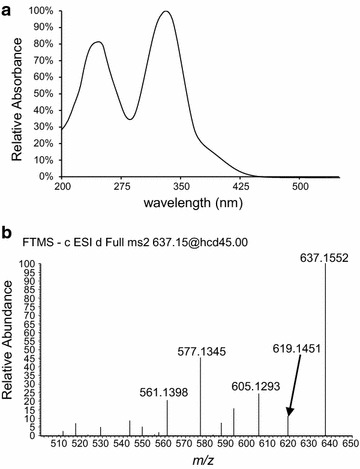
Characteristic UV (**a**) and MS/MS (**b**) spectra of the identified secalonic acid derivatives in the OE TF strain

In the OE TF cultures the red pigments endocrocin and clavorubin could be detected as well as prominent signals of the yellow ergochrome secalonic acid. Figure 7 clearly demonstrates difference in yellow pigment signals at UV 332 nm between the OE TF and the wild type. The marked signals at 34, 35 and 36 min correspond to a *m*/*z* value of 637.1554, matching closely to the theoretical mass 637.1561 for secalonic acid derivatives in the negative ionization mode. Furthermore, typical losses of water (*m*/*z* 619), a methoxy-group (*m*/*z* 605) and an add-on loss of CO_2_ (*m*/*z* 577) were obvious in the MS/MS spectra (Fig. 8). Unfortunately, the obtained information is insufficient to determine the specific derivate, as no references are commercially available. Nevertheless, three signals with the same UV spectra, exact mass and characteristic and synchronic MS/MS spectra correspond to all three different ergochromes.

**Fig. 9:**
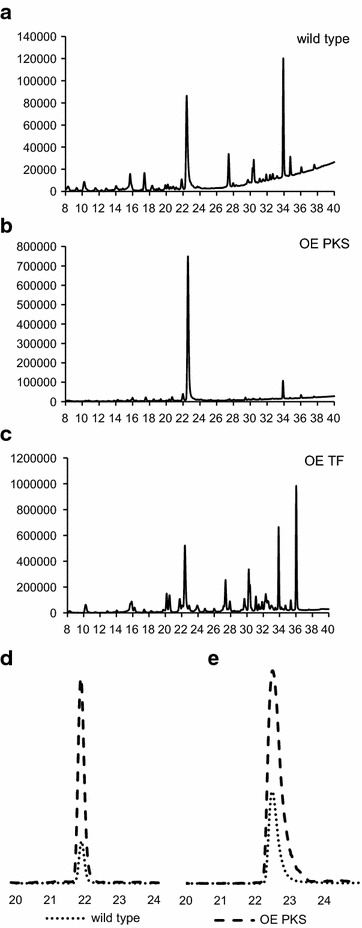
HPLC–DAD profiles of the wild type (**a**), the OE PKS (**b**) and the OE TF (**c**) as well as the HPLC–MS profile comparison of endocrocin (**d**) and clavorubin (**e**) between the wild type and OE PKS strain. Strains were grown under conditions unfavorable for the pigment production. The relative difference between the mass spectrometric signal intensity is represented

**Fig. 10:**
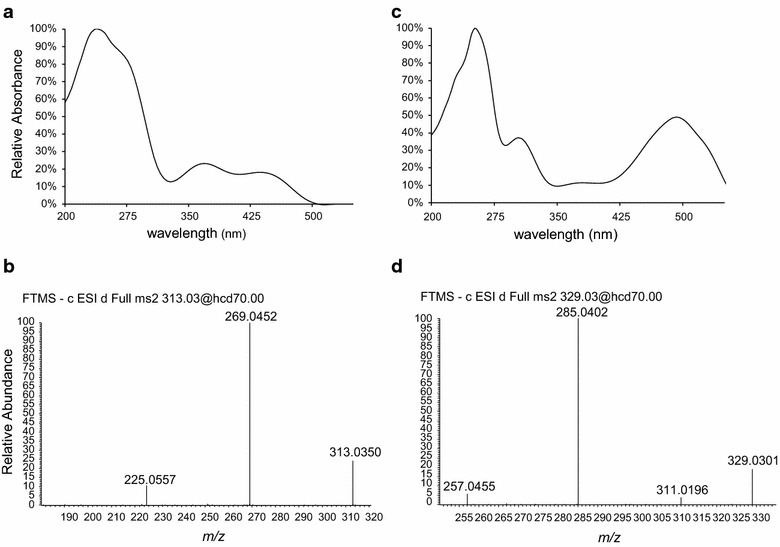
Characteristic UV and MS/MS spectra of the identified endocrocin (**a**, **b**) and clavorubin (**c**, **d**) in the OE TF strain

To search for anthraquinone derivatives, the DAD spectra were screened in the unique absorbance region of 440–480 nm for red pigments. The detailed UV spectra of the substances with a retention time of 21.8 and 22.8 min (Fig. 9a) strongly showed the characteristic absorbance maxima for endocrocin at a retention time of 21.8 min (Fig. 10a) as well as clavorubin at a retention time of 22.8 min (Fig. 10b) and therefore provided evidence for their occurrence. The UV signal itself is a compelling indicator of clavorubin as the principal anthraquinone metabolite with a much higher peak intensity compared to endocrocin. HRMS also unambiguously identified the peaks by comparing the experimental (313.0350 and 329.0301) and theoretical *m*/*z* values (313.0352 and 329.0301). The MS/MS experiments (Fig. 10d, e) additionally illustrated typical neutral losses of CO_2_ or water. In previous studies [23] those fragments were revealed as the most dominant and characteristic fragments for phenolic compounds, especially in the negative ionization mode.

To get further insight into the biosynthesis of the pigments, a strain where only the PKS *CPUR_05437* is overexpressed (OE PKS) was generated (Additional file 1: Figure S1B). Overexpression of the gene was confirmed by northern blot analyses (Additional file 1: Figure S1C) and the strain was cultured under growth conditions unfavorable for the pigment production. After 7 days of cultivation the culture was clearly pigmented. However, in contrast to the OE TF cultures the color was more purple red (Fig. 6). The UV signal of clavorubin at 22.8 min in Fig. 9 highlights the considerable production of the red pigments in the OE PKS strain (Fig. 9b) compared to the wild type (Fig. 9a). The UV intensity indicates an approximately 10 times higher production of clavorubin in the OE PKS mutant compared to the wild type and a two times greater production compared to the OE TF. In addition, Fig. 9d, e illustrates the increased mass spectrometric signal intensity of endocrocin, as well also of clavorubin, between the wild type and the OE PKS mutant.

Taken together, chemical analyses of the OE TF and the OE PKS cultures clearly show that activation of the whole cluster primarily leads to increased production of the yellow ergochrome dimers, whereas overexpression of the PKS merely increases occurrence of red anthraquinones.

### PKS CPUR_05437 is responsible for pigmentation in axenic culture and *in planta*

**Fig. 11:**
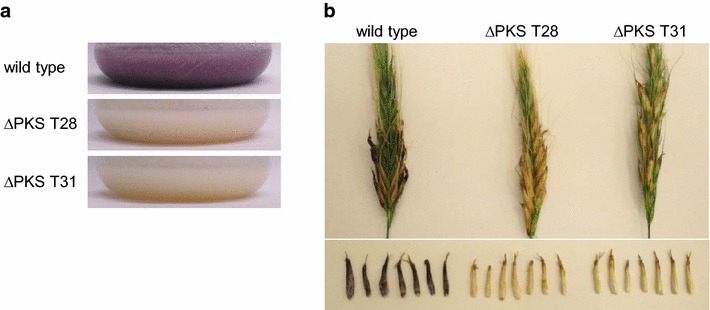
Knock out of the PKS leads to a loss of pigmentation. **a** The *C. purpurea* wild type as well as the two ∆*PKS* mutants T28 and T31 were grown for 10 days in liquid media containing 100 g/L sucrose and 4 g/L KH_2_PO_4_. **b** Rye plants were infected with the *C. purpurea* wild type as well as the ∆*PKS* mutants T28 and T31. Pictures were taken 5 weeks post-infection

Finally to prove that the PKS CPUR_05437 is the key enzyme in the pigment biosynthesis in *C. purpurea*, the corresponding gene was knocked out. Replacement of the gene by a phleomycin resistance cassette was verified by diagnostic PCR and Southern blot analysis (Additional file 2: Figure S2). Two independent knock out mutants as well as the *C. purpurea* wild type were grown in liquid media favorable for the pigment production (100 g/L sucrose and 4 g/L KH_2_PO_4_). After 10 days of cultivation the wild type culture had dark purple red pigmentation, whereas the cultures of the knock out mutants remained colorless (Fig. 11a).

**Fig. 12:**
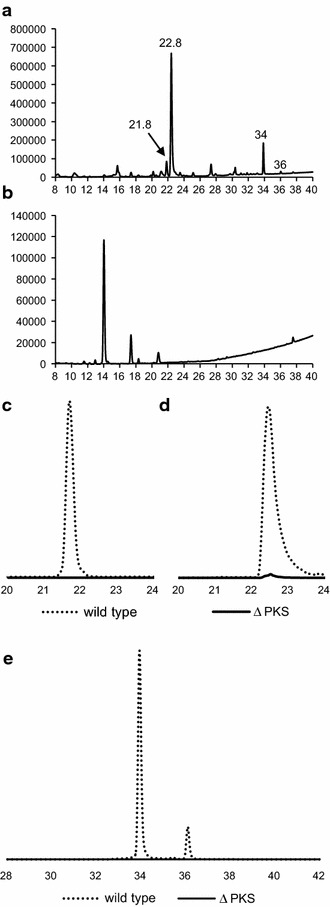
HPLC–DAD profiles of the wild type (**a**) and ∆*PKS* (**b**) strain, as well as the HPLC–MS profile comparison of endocrocin (**c**), clavorubin (**d**) and secalonic acid (**e**) between the wild type and ∆PKS strain. Strains were grown under conditions favorable for the pigment production. The relative difference between the mass spectrometric signal intensity is represented. The peaks in (**a**) are endocrocin (21.8 min), clavorubin (22.8 min) and two secalonic acid derivatives with a retention time of 34 and 36 min

To verify the visible impressions, the metabolite profiles of both strains were compared using RP–HPLC–DAD–HRMS. In the wild type cultures the red pigments endocrocin and clavorubin as well as the yellow secalonic acids were detected (Fig. 12a). In the cultures of the knock out mutants neither the red nor the yellow pigments were detectable in the DAD spectra (Fig. 12b) or in HRMS measurements (Fig. 12c–e).

To show that the PKS is also responsible for the typical pigmentation of the ergot sclerotia *in planta*, rye plants were infected with the *C. purpurea* wild type as well as the PKS knock out mutants. The pathogenicity assay shows that the mutants were able to infect the plants normally. First signs of successful infection (honeydew production) were evident 7–8 days post-inoculation. Approximately 15 days post-inoculation sclerotia were visible. After about 3 weeks the sclerotia of the wild type were pigmented purple black. In contrast, sclerotia of the ∆PKS mutants were colorless (Fig. 11b). Notably, sclerotia of all groups were of similar size and consistency. Complementation of the knock out with a PKS overexpression construct restored the pigmentation in axenic culture as well as *in planta* (Additional file 3: Figure S3).

Taken together the results clearly show that the PKS CPUR_5437 gene cluster is responsible for the formation of both kinds of ergot pigments; this applies both to the red anthraquinones and to the yellow ergochromes which are more complex xanthone derivatives.

## Discussion

Pigments are produced by a variety of filamentous fungi. In *Aspergillus flavus* or *Alternaria alternata*, for example, pigments such as melanin and asparasone play a protective role against abiotic stresses, e.g. UV radiation [24, 25]. In other fungi like the human pathogen *A. fumigatus* and the plant pathogen *Magnaporthe oryzae* melanin is required for virulence [26]. Pigmentation is often associated with developmental structures like spores or sclerotia. *C. purpurea* produces pigments mainly in the sclerotia. Sclerotia are resting structures and thus there is a strong requirement for them to be able to overcome biotic and abiotic stresses over a longer period in the field. Sclerotia of *A. flavus* asparasone minus mutants, for example, were significantly less resistant to insect predation and more susceptible to ultraviolet light and heat [25]. According to the reported functions of pigments in other fungi, it could be assumed that the ergot pigments are also important for the survival of the *C. purpurea* sclerotia. However, there is no difference in the consistency of the albino sclerotia in comparison to the wild type assuming that they are not more sensitive to mechanical damage. Interestingly, it has been shown that knock out of members of the NADPH oxidase (Nox) complex, usually involved in infection processes of pathogenic fungi, leads to the formation of white but small and immature pseudosclerotia in *C. purpurea* [27, 28]. qRT-PCR revealed a reduced PKS Cpur_05437 gene expression in the Δ*cpnox2* sclerotia [28] showing the complexity of pathogenic development and secondary metabolism in *C. purpurea*. Furthermore, the formation of the ergot pigments is contemporary with ergot alkaloid synthesis *in planta*. As the ergot alkaloids are light sensitive, pigmentation of the sclerotia is important for the protection of these toxins [29]. Surprisingly, in axenic culture, the expression of the pigment cluster and the alkaloid cluster are regulated contrary. While for the alkaloid production low levels of phosphate and a high sucrose concentration are necessary, the pigment production is increased by high levels of phosphate and repressed by high levels of sucrose. Apparently, there are different signaling pathways regulating the secondary metabolite production *in planta* and in axenic culture. Nevertheless, phosphate seems to be an important factor influencing the secondary metabolism of *C. purpurea*. As the alkaloid biosynthesis [30], the pigment biosynthesis is regulated by phosphate on a transcriptional level, but the molecular mechanism is still unknown. Generally, only little is known about the phosphate control of secondary metabolism. There are some examples that high concentrations of phosphate interfere with secondary metabolism of microorganisms [31]. Inorganic phosphate affects enzyme activities such as kinases and phosphatases, directly required in secondary metabolite biosynthesis or involved in signal transduction cascades, regulating e.g. fungal development, differentiation and other processes [32, 33]. Usually secondary metabolite production is repressed by high levels of phosphate like the production of the antifungal protein (AFP) by *Aspergillus giganteus* [34], the production of bikaverin by *Fusarium oxysporum* [35] or the aflatoxin production *Aspergillus parasiticus*. To our knowledge, the pigment cluster in *C. purpurea* is the first fungal secondary metabolite cluster which is induced by high levels of phosphate.

Interestingly, there are also differences when you compare between the expression levels of the cluster genes in the wild-type under inducing conditions (Fig. 5a) and the OE_TF strain under non-inducing culture conditions (Fig. 5b). The key gene, the PKS CPUR_5437 is highly expressed in both strains. However, some cluster genes like CPUR_5431, CPUR_5434 or CPUR_5435 show a considerable higher expression in the wild-type under inducing conditions than in the OE_TF strain under non-inducing conditions. Regulation of secondary metabolite clusters usually occurs on several levels. These results might indicate that at least for some of the pigment cluster genes, regulation by the culture conditions occurs on a higher level than the regulation by the transcription factor. However, it should be taken into account that Northern blots in Fig. 5a, b are two different experiments and therefore cannot be directly compared.

**Fig. 13:**
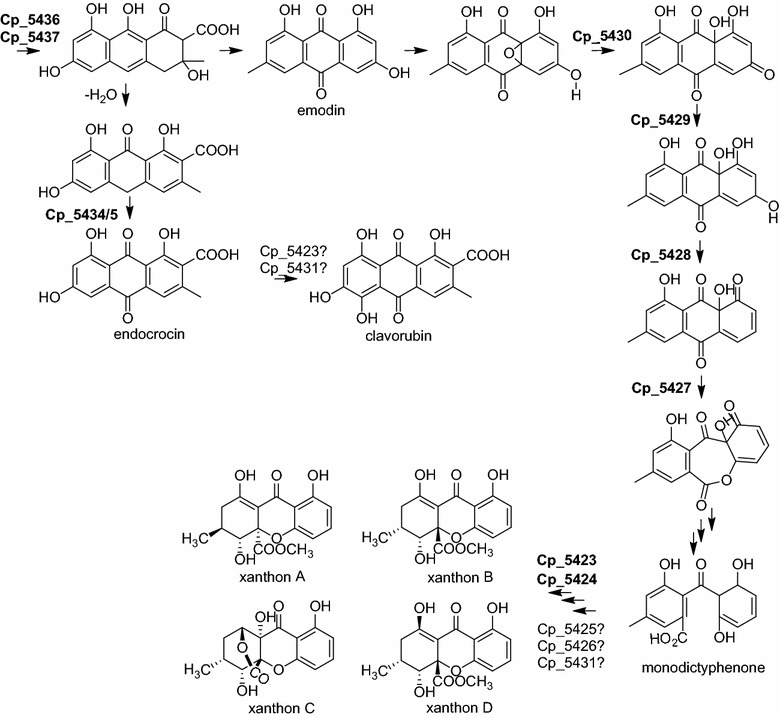
Proposed biosynthetic pathway of ergot pigments. The xanthone units of the yellow ergochromes and the red anthraquinone pigments endocrocin and clavorubin are products from the same PKS derived precursors. The PKS gene was deleted in this study, assignment of roles for all other enzymes in *bold* were made based on comparison to similar characterized biosynthetic pathways [12, 13, 19]. Roles for enzymes in regular font are due to the predicted function of the corresponding gene (Table 1)

Several classes of fungal pigments are described including carotenoids, melanins, flavins, and quinones [36, 37]. *C. purpurea* produces two groups of yellow to red pigments: anthraquinonecarboxylic acids and dimeric hydroxanthone derivatives. Several fungi are able to produce anthraquinone pigments, either as cluster end products or as intermediates or shunt products. In *A. fumigatus*, for example, endocrocin is a product of two different gene clusters [17, 19]. Ergochromes have also been isolated from other fungi [38] but no corresponding gene clusters have yet been characterized. First reported were the ergochromes in *C. purpurea* [39]. In the 1960s the structures of the ergot pigments, as well as of several intermediates of their biosynthesis, have been largely established [8]. Here we report on the pigment gene cluster in *C. purpurea* for the first time and show that both groups of ergot pigments are products of the same biosynthetic pathway. Based on these results, the knowledge about pathway intermediates [8] and on comparison to similar characterized biosynthetic pathways [12, 17, 19] a tentative biosynthetic pathway of the ergot pigments is proposed (Fig. 13).

Most likely endocrocin and clavorubin are shunt products in the pathway of xanthone biosynthesis. In the next steps dimerization of different xanthone units would lead to the formation of ergochromes. Genes *CPUR_05425*, *CPUR_05426* and *CPUR_05431* are unique to *Clavicep*s in comparison to the *A. nidulans* xanthone cluster. Thus it is likely that these genes are involved in further modification of xanthone units or in their dimerization. Moreover, the metabolite profile of the OE TF strain (Fig. 7) shows several new peaks where the corresponding *m*/*z* value does not fit with any known metabolite. Further investigations of these peaks may lead to the discovery of so far unknown ergochromes of *C. purpurea*.

Another interesting aspect is that single overexpression of the cluster key gene, the PKS CPUR_5437, is sufficient to induce production of the red anthraquinone pigments. As the cluster is not completely silent under these culture conditions, the expression level of the other cluster genes seems to be enough for the production of the simple pigments endocrocin and clavorubin but not for the complex structures of the ergochromes. It also might be that the increased through-put of the first pathway intermediate leads to a feedback loop and an up-regulation of other pathway genes.

Activation of the pigment cluster in axenic culture opens up new possibilities for uncovering the full biosynthetic pathway and for genetic engineering of the metabolic pathway to improve pigment production, or even to obtain modified molecules with novel bioactivity. Pigments are used as dyes for textiles, in cosmetics and as food colorants. Anthraquinone pigments, like the ones produce by *C. purpurea*, have several advantages; for example, they are relatively stable and have good light-fastness and brightness [40]. There is increasing commercial interest in the production of pigments by filamentous fungi as sources of cheaper, more ecologically friendly alternatives to existing dyes. Thus fungal pigment synthesis has several advantages over chemical methods [36, 41]. Besides their role as colorants, there is also a pharmaceutical value of ergot pigments, especially of the ergochromes. Ergoflavin, for example, has anti-inflammatory and anticancer properties [10]. Secalonic acid A shows cytostatic and anti-tumor activity [9] and, additionally, has a neuroprotective effect, making it to an interesting possibility for the treatment of neurodegenerative diseases [11].

## Conclusions

This study reports the identification and characterization of the ergot pigment gene cluster in *C. purpurea*. This gene cluster has been shown to be responsible for the typical purple-black color of ergot sclerotia but is not required for pathogenicity of the fungus. Furthermore, knock out of the pathway key enzyme, a NR-PKS, finally proved that both groups of ergot pigments, the red anthraquinonecarboxylic acids and the yellow ergochromes, are products of the same biosynthetic pathway. Fungal pigments gain increasing interest as ecologically friendly dyes or for the development of new pharmaceuticals. Activation of the *C. purpurea* pigment gene cluster in axenic culture and optimization of the culture conditions opens up new possibilities for biotechnological applications.

## Methods

### Strains and culture conditions

*Claviceps purpurea* strain Ecc93 has been described previously [42]. Mycelia were grown on BII medium [43] for maintenance and DNA isolation or on Mantle medium for conidia harvesting [44]. For secondary metabolite production strains were cultivated on a rotary shaker at 26 °C in modified media according to Amici [45] with sucrose and PO_4_ concentrations as indicated.

Yeast strains FY834 [46] used for the yeast recombinational cloning method were incubated at 30 °C in yeast extract-peptone-dextrose (YPD) or in synthetic dextrose (SD) medium lacking the selecting amino acids.

### Chemical and materials

All chemicals were purchased from Sigma-Aldrich GmbH (Seelze, Germany), Carl Roth GmbH + Co. KG (Karlsruhe, Germany), or VWR International GmbH (Darmstadt, Germany). Solvents were obtained in gradient grade quality. Water for HPLC was purified by a Milli-Q Gradient A 10 system (Millipore, Schwalbach, Germany).

### Nucleic acid extraction and analysis

Genomic DNA from *C. purpurea* was isolated as described by Cenis [47]. For Southern blot analysis, 5–10 μg of digested genomic DNA were separated via gel electrophoresis in a 1 % agarose gel with salt-free buffer [47] and transferred to a nylon membrane (Nytran SPC; Whatman). For the isolation of RNA, the RNAgents total RNA isolation kit (Promega GmbH, Mannheim, Germany) was used. For northern blotting, 20 mg RNA were used for the separation on a 1 % (w/v) agarose gel containing 1 % (v/v) formaldehyde and afterwards transferred to a nylon membrane (Nytran SPC; Whatman). For southern as well as northern hybridization, 32P-labeled probes were generated using the random oligomer-primer method, and hybridized to the membranes. PCR reactions were performed using either the BioTherm Taq DNA Polymerase (GeneCraft, Germany) or the proof reading Phusion DNA polymerase (Finnzymes, Finland). Primers were synthesized by Biolegio (Nijmegen, Netherlands).

### Vector construction

Vectors were constructed using the yeast recombinational cloning method [48], based on the described vector system [49, 50]. The sequences of all primers used are listed in Additional file 4: Table S1. For construction of the *cp5433* overexpression vector, the *cp5433* gene was amplified with Phusion polymerase with the primers OE_Cp5433_F and OE_Cp5433_R from genomic DNA and recombined with the NotI-NcoI-digested pNAH-OGG vector [50]. For construction of the *cp5437* overexpression vector, the gene *cp5437* was amplified with the primers OE_PKS4_F and OE_PKS4_R1 and OE_PKS4_F1 and OE_PKS4_R from genomic DNA using Phusion polymerase and recombined with the NotI-NcoI-digested pNAH-OGG vector [50].

For construction of the *cp5437* replacement vector, the flanking regions of *cp5437* were amplified with the primers PKS4_5F and PKS4_5R for the 5′ flank as well as PKS4_3F and PKS4_3R for the 3′ flank. Primers contain overlapping sequences toward the yeast shuttle-vector pRS426 or the phleomycin resistance cassette. The phleomycin resistance cassette was amplified with the primers CpBle1F and CpBle1R from pRS426CpBle. The yeast shuttle vector pRS426 [48] was linearized by restriction with XhoI and EcoRI.

For homologous recombination the vector fragments were transformed into yeast strain FY834. The resulting vectors were selected on SD medium lacking uracil. DNA was isolated from yeast cells using the SpeedPrep yeast plasmid isolation kit (DualSystems) and transformed into *Escherichia coli* TOP10’ for amplification.

### Fungal transformation

Protoplasts of *C. purpurea* were generated with lysing enzymes from *Trichoderma harzianum* (Sigma-Aldrich, St. Louis) and transformed with 10 μg of vector DNA as described by Jungehülsing et al. [51]. For selection, either phleomycin was directly applied to the protoplasts (33 μg/mL) or hygromycin was applied to regenerated protoplasts 24 h after transformation by overlay agar (1.5 mg/mL). Resistant colonies were transferred to fresh selective medium (BII, pH 8, 100 μg/mL phleomycin or 0.5 mg/mL hygromycin). By PCR using specific primers as indicated, resistant transformants were checked for the integration of the vector.

### Pathogenicity assays

Male sterile rye plants (*Secale cereale*) were cultivated in growth chambers as described by Smit and Tudzynski [52]. Florets of blooming ears (30–40 florets per ear) were inoculated with 5 µL of a suspension containing 2 × 10^6^ conidia/mL as described by Tenberge et al. [53]. Afterwards, the ears were covered with paper bags equipped with cellophane windows to avoid cross contamination.

### qRT-PCR

For reverse transcription of the RNA template, Superscript II reverse transcriptase (Invitrogen, Darmstadt, Germany) was used. Real-time qPCR reactions were performed with the Bio-Rad iQ SYBR Green Supermix and the iCycler Thermal Cycler (Bio-Rad, Hercules, CA, USA). iCycler iQ Real-Time Detection System Software (version 3.0; Bio-Rad) was used for programming, data collection, and analyses. Expression of *cp5436* was detected by the primers RTq_LN4_F and RTq_LN4_R and normalized to the expression of the housekeeping genes β-tubulin (CCE34429.1), γ-actin (AEI72275.1), and glyceraldehyde-3-phosphate dehydrogenase (X73282.1) [27] using primers Actin_uni and Actin_rev, Tub_uni and Tub_rev, and Gpd_uni and Gpd_rev.

### Analysis of fungal mycelium

The mycelium was extracted with a mixture of acidified ethyl acetate and water. Water (3 mL) and of organic solvent (4 mL) were added to the mycelia in a 15 mL tube and shaken for 45 min. In the next step, phases were separated and the organic solvent evaporated under nitrogen at 30 °C. Residue was dissolved in 700 µL acetonitrile/water 1/9 (v/v) and 15 µL used for HPLC injection.

### RP–HPLC–DAD–HRMS measurements

In order to identify the pigments, the extracted mycelia were measured by RP–HPLC–DAD–HRMS. For the HRMS measurement, an Accela LC 60057-60010 system (Thermo Fisher Scientific, Bremen, Germany) was linked to a LTQ Orbitrap XL mass spectrometer (Thermo Fisher Scientific). A SPD-M20A Shimadzu PDA Detector (Shimadzu, Duisburg, Germany) was coupled to the MS spectrometer. Data acquisition was performed with Xcalibur 2.07 SP1 (Thermo Scientific). Separation was carried out on a 150 × 2 i.d., 3 μm, ReproSil-Pur C18-AQ (Dr. Maisch GmbH, Amerbuch, Germany) using a binary gradient at a column temperature of 40 °C. The injection volume was 15 µL and the autosampler was cooled to 7 °C. The flow rate was set to 260 µL/min. Solvent A was acetonitrile with 0.1 % of formic acid (v/v) and solvent B was water with 0.1 % of formic acid (v/v). The HPLC was programmed as follows: in the first 4 min isocratic 15 % of A, afterwards a binary gradient to 40 % in 21 min and next during 16 min up to 100 % of A. Then the column was washed with 100 % A and equilibrated at starting conditions. For the detection of the expected pigments a total ion scan of a mass range from *m*/*z* 165–950 with a resolution of 60,000 in the negative ion mode was used. To confirm the substances, subsequent mass spectrometric fragmentation experiments in the negative mode were used. The experiments included high-energy collision dissociation (HCD) with a relative energy of 40–85 %, depending on the ionization and an isolation width of *m*/*z* 1.5 with and activation time of 30 ms. The fragments were analyzed with the Orbitrap detector at a resolution of 30,000.

### Mass spectrometer and DAD parameters

The LTQ Orbitrap XL was used with a heated electrospray ionization technique. The sheat gas flow was 30 arbitrary units, the aux gas flow 15 arbitrary units and the sweep gas flow 10 arbitrary units. In the negative mode, vaporizer temperature was set to 300 °C and capillary temperature to 270 °C. The source voltage was 3.0 kV, capillary voltage −33 V and Tube Lens −160 V.

The Shimadzu PDA-Detector had the following parameters: starting wavelength 200 nm, ending wavelength 700 nm, with a wavelength step of 4 nm. The sampling frequency was consequently 4.16 Hz.

## Availability of supporting data

The data sets supporting the results of this article are included within the article and its additional files. Gene sequences are available online: http://pedant.gsf.de/pedant3htmlview/pedant3view?Method=analysis&Db=p3_p76493_Cla_purpu.

## Additional files

**Additional file 1: Figure S1.** Generation of *Cpur_05433* and *Cpur_05437* overexpression mutants.

**Additional file 1: Figure S2.** Generation of *Cpur_05437* knock out mutants.

**Additional file 1: Figure S3.** Complementation of the *Cpur_05437* knock out.

**Additional file 4: Table S1.** Oligonucleotide primers used in this study.
