# Supplementary material for: Identification and characterization of the ergochrome gene cluster in the plant pathogenic fungus Claviceps purpurea
Source: Fungal Biol Biotechnol. 2016 Mar 22;3:2. doi: 10.1186/s40694-016-0020-z (PMC5611617; doi:10.1186/s40694-016-0020-z)
Supplement: Supplementary file 1 — Additional file 1: Figure S1. Generation of Cpur_05433 and Cpur_05437 overexpression mutants. [file 40694_2016_20_MOESM1_ESM.pdf]

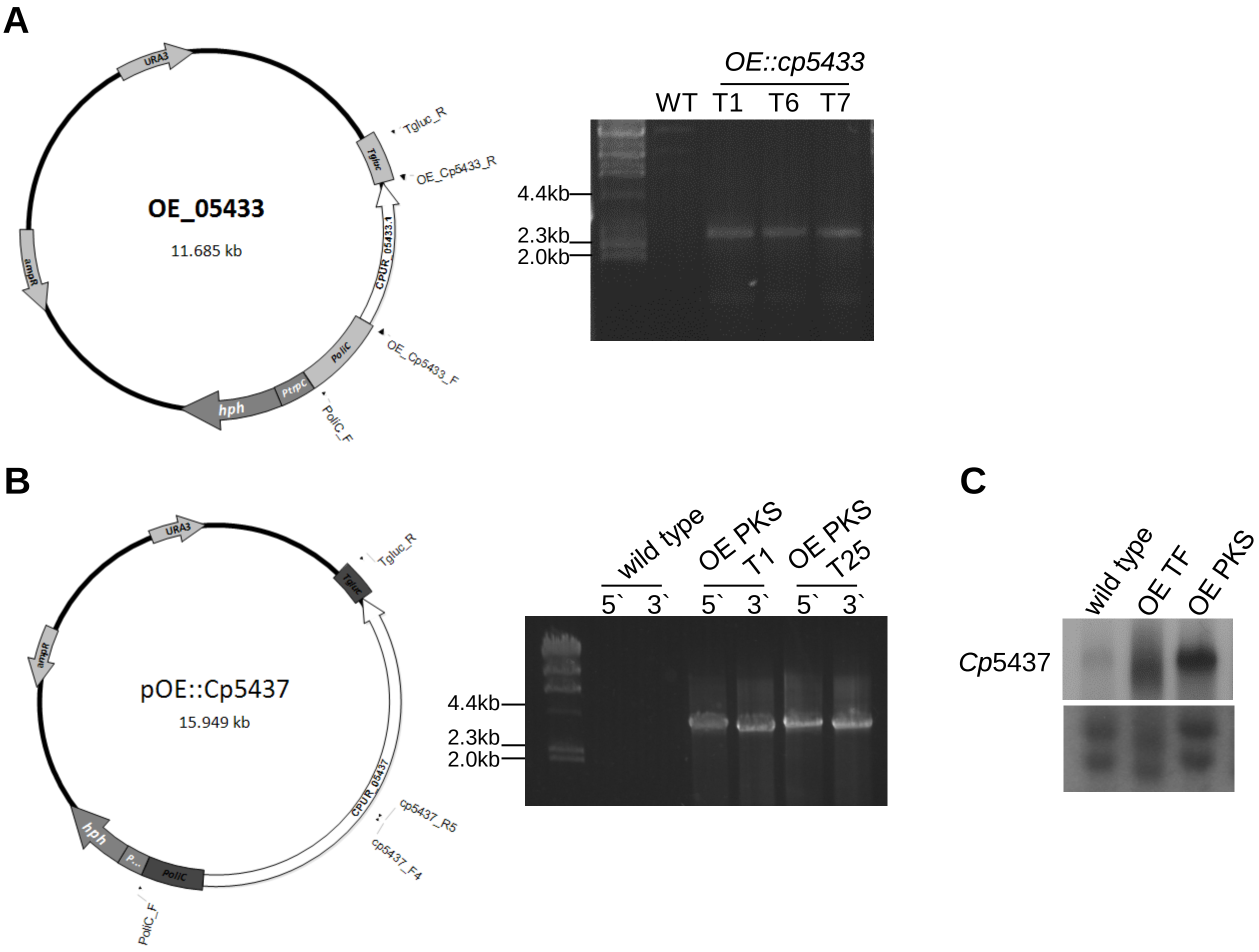

Figure S1: Generation of *Cpur_05433* and *Cpur_05437* overexpression mutants

A) Verification of genomic presence of the *Cpur_05433* overexpression construct. Transformants with ectopic integration of the overexpression vector were identified via PCR using primer pair PoliC_F and Tgluc_R.

B) Verification of genomic presence of the *Cpur_0*5437 overexpression construct. Transformants with ectopic integration of the overexpression vector were identified via PCR using primer pairs PoliC_F and cp5437_R5 and Tgluc_R and cp5437_F4.

C) Verification of Cpur_05437 overexpression by northern analysis
