# Supplementary material for: Identification and characterization of the ergochrome gene cluster in the plant pathogenic fungus Claviceps purpurea
Source: Fungal Biol Biotechnol. 2016 Mar 22;3:2. doi: 10.1186/s40694-016-0020-z (PMC5611617; doi:10.1186/s40694-016-0020-z)
Supplement: Supplementary file 2 — Additional file 1: Figure S2. Generation of Cpur_05437 knock out mutants. [file 40694_2016_20_MOESM2_ESM.pdf]

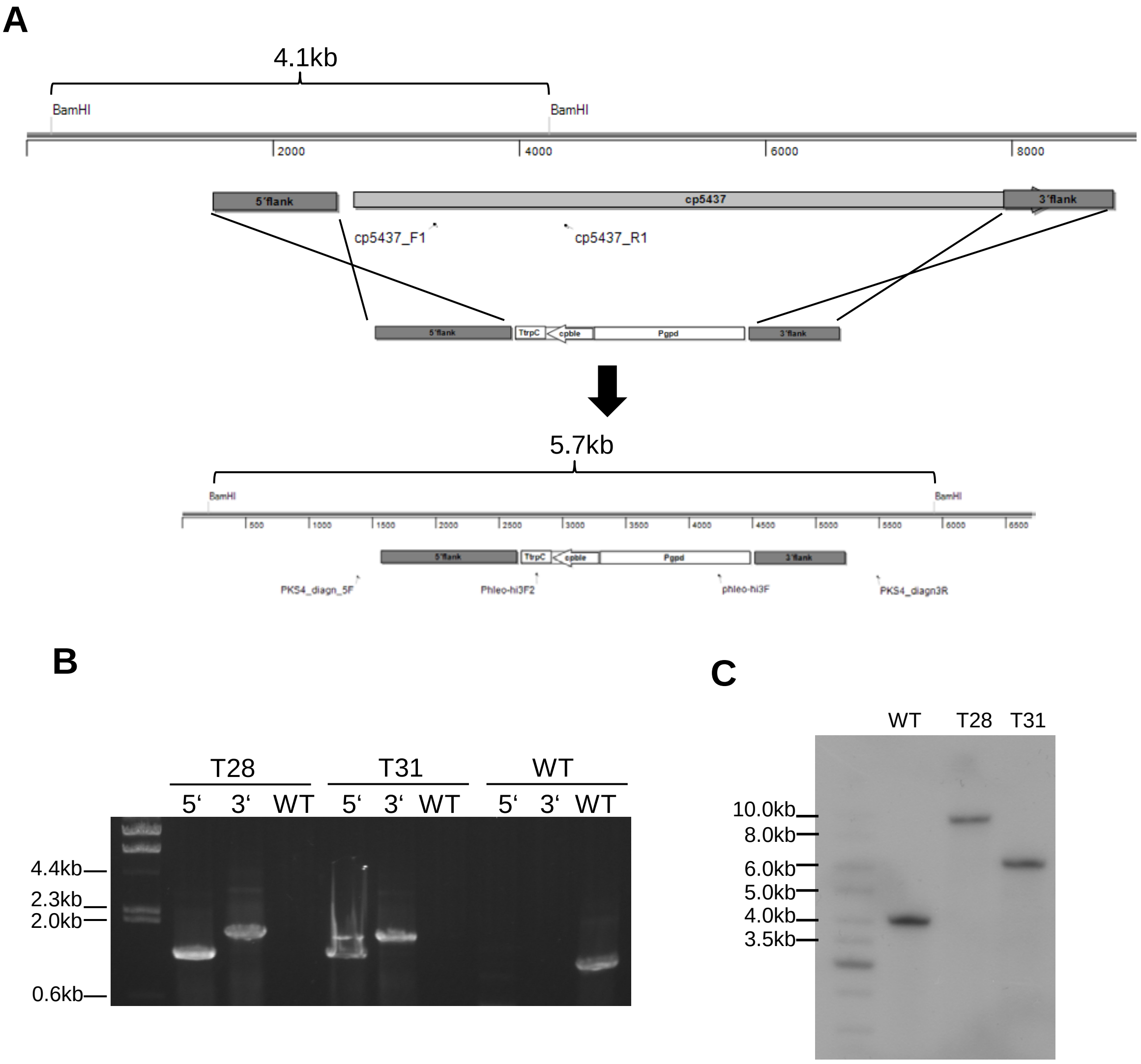

Figure S2: Generation of *Cpur_0*5437 knock out mutants.

A) Deletion strategy. Knock out mutants were generated by homologous integration of a phleomycin resistance cassette via a double cross over event. Primers for diagnostic PCR as well as restriction sites for southern blot analysis are indicated.

B) Diagnostic PCR. Homologous integration of the knockout vector was verified by amplification of the 5´ diagnostic fragments using primer pair PKS4_diagn_5F and Phleohi3F2 and the 3´ fragment using primer pair PKS4_diagn_3R and Phleohi3F. Lack of the wild type control fragment shows the absence of the gene cp5437.

C) Southern blot analysis. Genomic DNA of the two independent Cpur_05437 knockout mutants T28 and T31 as well as of the wild type was digested with BamHI. As a probe for southern blot the 5’ flank of *Cpur_0*5437 was used.
