# Supplementary material for: Identification and characterization of the ergochrome gene cluster in the plant pathogenic fungus Claviceps purpurea
Source: Fungal Biol Biotechnol. 2016 Mar 22;3:2. doi: 10.1186/s40694-016-0020-z (PMC5611617; doi:10.1186/s40694-016-0020-z)
Supplement: Supplementary file 3 — Additional file 1: Figure S3. Complementation of the Cpur_05437 knock out. [file 40694_2016_20_MOESM3_ESM.pdf]

A

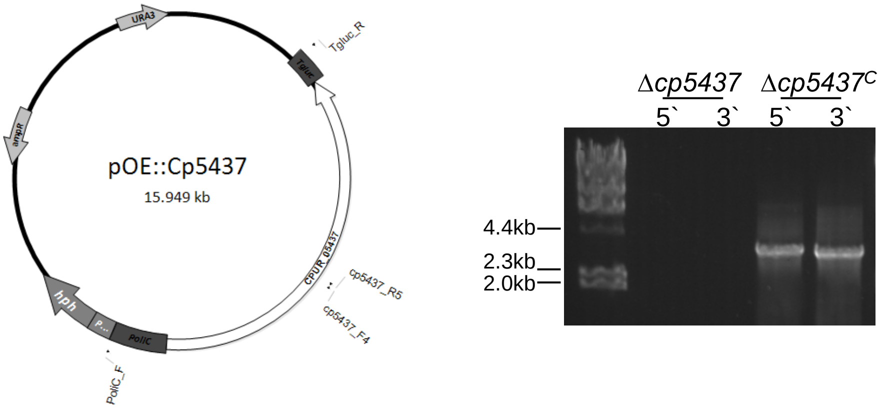

B

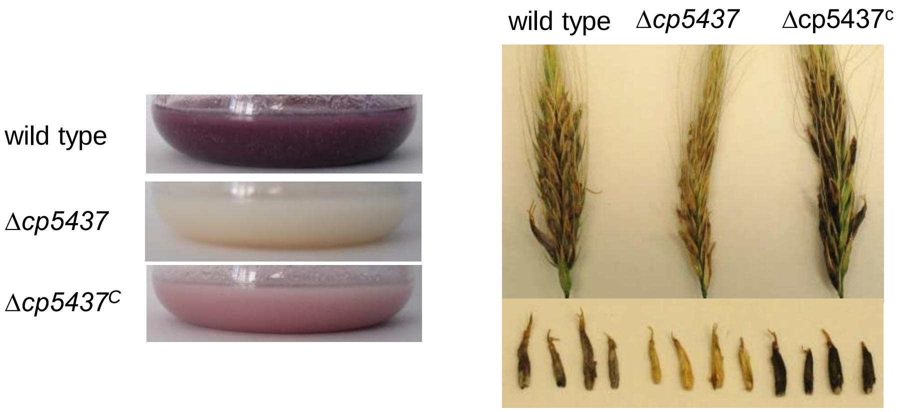

Figure S3: Complementation of *Cpur_05437* knock out mutant T28

A) Verification of genomic presence of the *Cpur_05437* overexpression construct. Transformants with ectopic integration of the overexpression vector were identified via PCR using primer pairs PoliC_F and cp5437_R5 and Tgluc_R and cp5437_F4.

B) Restoration of phenotype in the Δ*Cpur_05437*^{C} strain in axenic culture and *in planta*.
