# Supplementary material for: Identification and characterization of the ergochrome gene cluster in the plant pathogenic fungus Claviceps purpurea
Source: Fungal Biol Biotechnol. 2016 Mar 22;3:2. doi: 10.1186/s40694-016-0020-z (PMC5611617; doi:10.1186/s40694-016-0020-z)
Supplement: Supplementary file 4 — Additional file 4: Table S1. Oligonucleotide primers used in this study. [file 40694_2016_20_MOESM4_ESM.pdf]

Table S1: Oligonucleotide primers used in this study

Name	Sequence (5'-3')
**Overexpression vector construction**	
OE_Cp5433_F	CATCACATCACAATCGATCCAACCATGGACCATTCCATTGGCG
OE_Cp5433_R	CATCTTATCTACATACGCTAAGCCTACAATCTTCGCAGCATGT
OE_PKS4_F	CATCACATCACAATCGATCCAACCATGGCGATCTATACCCCGAAC
OE_PKS4_F1	GCACAGCTGGCGCGTGGCGATGG
OE_PKS4_R	TAATCATACATCTTATCTACATACGTCAGCTATAATATTCCTCGAGC
OE_PKS4_R1	CCATCGCCACGCGCCAGCTGTGC
**Knock out vector construction**	
PKS4_5F	CCAGGGTTTTCCCAGTCACGAGGATCCTACGATCTATCTCTGGCACC
PKS4_5R	CACTTAACGTTACTGAAATCCGAGCTTCTTCAATGCAACG
PKS4_3F	TCCTTCAATATCATCTTCTGTCTTCAGCACCAGGCAAGTCC
PKS4_3R	ACAATTTCACACAGGAAACAGCGGATCCCCACGAAATCTACCAGACAGG
CpBle1F	CGGAGACAGAAGATGATATTGAAGGAGCGATCGAGACCTAATACAGCCCC
CpBle1R	GTTGGAGATTTCAGTAACGTTAAGTGGGCATTGCAGATGAGCTGTATCTG
**Diagnostic PCRs**	
PKS4_diagn5F	ATCTGCTCAACGTCTGGCAGC
PKS4_diagn3R	GTTAGGTATAGGCGCACTTTGG
Phleohi3F2	GTGTTCAGGATCTCGATAAGATACG
Phleo-hi3F	GGCTCAAGTCATGACCCTCTGGG
cp5437_F1	TGTCAGAGCTGCTTGAAGC
cp5437_R1	CACCTCACGATAATCGTCC
PoliC_F	GTCAAGATTTGCGTCCGAGG
Tgluc_R	CATATGGTAACGCCACATGAGG
cp5437_F4	CCTCACATCGGCATAAAGG
cp5437_R5	GAATGTGTAGATTGCGCTTCG
**Expression studies (qRT PCR)**	
Actin uni	GCCGTTTTCCCCTCTATCGTC
Actin rev	ACATACGAGTCCTTCTGACCCAT
Tub uni	TACAATGGTACCTCGGAGCAAC
Tub rev	CCAGAGGCCTCATTGAAGTAGAC
Gpd uni	CCCGAATATGCTGCCTACATGCT
Gpd rev	CGTCCTTCTTGATCTCGCCCT
RTq_LN4_F	GGCCAAAATCCAGGCAAGTTCAC
RTq_LN4_R	ACTCCACCGGTGTGATCGC
**Expression studies (northern)**	
cp5422.1 F1	AACACCGAGCCACCGAAAGC
cp5422.1 R1	ATCTGAACGGCTATGAATGC
cp5423.1 F1	ACGAAGCAGCGATGCTTGG
cp5423.1 R1	TCACGACCAATCTCCATCC
cp5424.1 F1	ATGGAATCGACCATCCCAGC
cp5424.1 R1	CTACTCCACACTCGCGTCAG
cp5425.1 F1	ATGCCTGCCCCTGCCGAGG
cp5425.1 R1	TCGCCGTGGGAGGCTGTCC
cp5426.1 F1	ATGCCTGCCCCTGTCGAG
cp5426.1 R1	CTAAGAACCTGCCACCGC
cp5427_F1	ATGGCATCAACTACGGGAAC
cp5427_R1	TCGAGCCTTCCACTCAACC
cp5428_F1	TCACTCCGCAAAGATTTTGTCG
cp5428_R1	ATGTCTAAAACTCCCTCGTACG
cp5429_F1	ATGTCGGAAACTCACATTCC
cp5429_R1	TCATGCAGCTCCTCCATCC
cp5430_F1	ATGGCCTCATACGCAGTGC
cp5430_R1	TTACGGCAAATACGGATGTAG
cp5431_F1	ACATCGAATGCGAGCTCTCC
cp5431_R1	CACAGAACATGACAAGTATCC
cp5432_F1	TGGCTTGCATACCTCTCAGC

cp5432_R1	CATCTGATACATGATCAATCC
cp5434_F1	ATGAAGATGGGCATGGCCAAG
cp5434_R1	CTACACGGCCTTTCCATCC
cp5435_F1	ATGCTCCGAGGGTTTGCC
cp5435_R1	TCAGTTCGCGGATCCGACG
cp5436_ 5F	GTGACAAGGGCGGATATAGG
cp5436_ 3R	CTACTCTACAGAGTACCACCG
cp5437_F1	TGTCAGAGCTGCTTGAAGC
cp5437_R1	CACCTCACGATAATCGTCC
cp5438_F1	ATGGAACCAGCCACGAGAG
cp5438_R1	CTATGTCTGAGCGCAGGCTG
